# Atherosclerotic Renal Artery Stenosis: A Review

**DOI:** 10.1055/s-0041-1730004

**Published:** 2021-10-12

**Authors:** Thilina Gunawardena

**Affiliations:** 1 Department of Vascular and Transplant Surgery, National Hospital of Colombo, Sri Lanka

**Keywords:** atherosclerosis, renal artery stenosis, revascularization in renal artery stenosis

## Abstract

Renal artery stenosis (RAS) is associated with hypertension and renal impairment. Atherosclerosis is the leading etiologic factor which accounts for >90% of the cases. Those with atherosclerotic RAS (ARAS) tend to have concomitant atherosclerosis in other vascular beds, so they are at a high risk of adverse coronary and cerebrovascular events. Management of ARAS is controversial, with limited indications for revascularization. In this review, the author aims to discuss the pathophysiology, natural history, diagnosis, and management of ARAS.

## Introduction

Renal artery stenosis (RAS) is characterized by narrowing of one or more main renal arteries or its branches. 1 Atherosclerosis is the leading cause and accounts for >90% of cases. Fibromuscular dysplasia is the second commonest cause. Vasculitis, neurofibromatosis, radiation, and external compression of the renal artery in retroperitoneal fibrosis are other less common causes. 2

Atherosclerotic RAS (ARAS) usually affects the renal artery ostia, proximal one-third of the main renal artery and the adjacent aorta. This contrasts with RAS associated with fibromuscular dysplasia where the distal two-thirds of the renal arteries tend to be involved. 2 In advanced cases of ARAS, intrarenal branch arteries can be affected as well. 3 In 20% with ARAS bilateral renal artery involvement or involvement of a single functioning kidney will be seen. 4

The exact incidence of ARAS in the general population is unknown as the majority with this condition will remain asymptomatic. 1 There seems to be a substantial risk of ARAS in those with atherosclerosis-related vascular disease elsewhere in the body. In patients with coronary artery disease, the prevalence of ARAS is estimated to be 11 to 23%. 5 In a cohort of patients who underwent digital subtraction angiography (DSA) for lower limb claudication or tissue loss, 44.9% had concomitant RAS. 6 According to the findings of an autopsy study by Kuroda et al, 7 12.1% with ischemic stroke were noted to have ARAS. As diabetes is a well-known risk factor for accelerated atherosclerosis, the incidence of ARAS is expected to be high in those with diabetes. Another autopsy study by Sawicki et al 8 reported that 8.3% of those with diabetes had ARAS. Due to associated atherosclerotic disease in the coronary and cerebral vascular beds, those with ARAS have a high risk of cardiovascular events. This was noted in the study by Kalra et al 9 where those with ARAS had a significantly higher risk of atherosclerotic heart disease, heart failure, cerebrovascular events, and chronic kidney disease.

ARAS is a progressive disease. In the study done by Zierler et al, 10 23% of those with <60% stenoses had progressed to 60% or more by end of the first year. This proportion increased to 42% by 2 years. Five percent of those with more than 60% stenosis at diagnosis progressed to complete occlusions by 1 year. This was 11% at the end of 2 years.

Ischemic nephropathy or loss in renal mass and deterioration of renal function is a well-known sequel of ARAS. According to a study by Strandness, 11 19% of patients with ≥60% stenosis of the renal arteries had a >1-cm loss in renal length at the end of first year. High-grade stenoses, elevated systolic blood pressure, and low velocity flow in the renal cortex on duplex were identified as risk factors for reduction of renal mass. 11 12 The exact contribution of ARAS as a cause for end-stage kidney disease is unknown. 13 According to Mailloux et al, 14 12% of patients who were referred for dialysis had ARAS as the predisposing cause for the renal failure.

Life expectancy is reduced in those with ARAS when compared with the general population. This seems to be true when patients with ARAS are compared with well-matched patients with essential hypertension. 5 Death is usually due to cardiovascular events. The mortality is even higher when ARAS is associated with end-stage renal failure. 4

## Pathophysiology

Reduction in renal perfusion due to RAS activates the renin–angiotensin system and leads to increased production of angiotensin II. Angiotensin II is a powerful vasoconstrictor and potentiates the vasoconstrictor effects of noradrenaline. It stimulates the production of aldosterone which increases the circulating blood volume by promoting fluid retention. Hypertension in RAS is attributed to these effects of vasoconstriction and increased circulatory volume. When the hypertension is long lasting, plasma renin activity decreases. Hence plasma renin levels cannot be used as a sensitive indicator to rule out renovascular hypertension. 2

## Presentation

Hypokalemia, presence of an abdominal bruit, and diagnosis of hypertension at an unusually young age are some classic features associated with RAS. 2 Acute onset decompensated heart failure, also known as flash pulmonary edema, is a presentation that is usually associated with bilateral RAS or RAS affecting a solitary functioning kidney. 15 American Heart Association (AHA) guidelines describe several clinical scenarios where RAS should be suspected and excluded. 13 Six scenarios are given below:

1. Onset of hypertension before 30 years of age.
2. Onset of severe hypertension after 55 years of age.
3. Accelerated hypertension (sudden, persistent worsening of previously controlled hypertension), resistant hypertension (hypertension not controlled with a three drug regimen which includes a diuretic), and malignant hypertension (hypertension with acute end organ damage).
4. New onset renal dysfunction or worsening of renal function after starting therapy with an angiotensin-converting enzyme inhibitor (ACEI) or angiotensin receptor blocker (ARB).
5. Unexplained atrophic kidney or discrepancy in the size of the two kidneys >1.5 cm.
6. Sudden, unexplained pulmonary edema (especially in patients with azotemia).

## Diagnosis

Once RAS is suspected, imaging studies should be done to confirm the diagnosis. DSA is the gold standard but it is invasive. Duplex, computed tomography angiography (CTA) and magnetic resonance angiography (MRA) are noninvasive imaging modalities.

## Duplex

Duplex ultrasound is cheap, readily available, and free of radiation. However, operator dependency is a disadvantage. Obesity and overlying bowel gas may interfere with proper image acquisition. There is a chance that accessory renal arties may be missed on duplex. 3 13 When compared against DSA, duplex has a sensitivity of 84 to 92% of and a specificity of 64 to 99% for detecting RAS. It is useful for follow-up imaging after intervention as well. 13

Spectral broadening and increased velocities are indicators of hemodynamically significant stenoses on duplex. When the ratio between renal artery peak systolic velocity (PSV) and aortic PSV (renoaortic velocity ratio) is >3.5, it indicates a 60% stenosis. Renal artery PSVs of >150 and 180 cm/s are suggestive of 50 and 60% stenoses, respectively. 3

Renal artery resistive index (RI; PSV − end diastolic velocity/PSV) can be calculated using duplex, and a high RI is indicative of renal microvascular disease. 3

## Computed Tomography Angiography

CTA involves exposure to radiation and iodinated contrast material. There is a risk of allergic reactions and contrast-induced nephropathy associated with intravascular contrast injection. CTA has the ability to acquire excellent images of the renal arteries, especially after three-dimensional reconstruction. Against DSA, it has a sensitivity of 59 to 96% and a specificity of 82 to 99% for detection of RAS. 3

## Magnetic Resonance Angiography

MR imaging (MRI) does not expose the patient to radiation or iodinated contrast. Gadolinium-based contrast material is used for image enhancement, and those with impaired renal function have a risk of nephrogenic systemic fibrosis when exposed to gadolinium. Claustrophobia and certain MRI incompatible, implantable devices may preclude MRA. 3 Compared with DSA, MRA has a sensitivity of 90 to 100% of and a specificity of 76 to 94% for diagnosing RAS.

## Digital Subtraction Angiography

DSA is invasive, and arterial puncture can be associated with risks such as bleeding, dissection, distal embolism, and pseudoaneurysm formation. Additionally, it exposes the patient to radiation and iodinated contrast as well. Considered the gold standard of imaging in RAS, DSA is rarely used as the preferred first line imaging modality due to the availability and advances in noninvasive techniques described above. According to the AHA guidelines, there is a place for DSA as a diagnostic tool in RAS when (1) images cannot be obtained by other noninvasive modalities, and (2) when the patient requires concomitant peripheral or coronary angiography for other indications.

Captopril renal scintigraphy, selective renal vein renin studies, plasma renin activity, and the captopril test are not recommended as diagnostic tests for RAS due to their poor sensitivity and specificity. 13

## Management

### Medical Therapy

There is a general consensus that all with ARAS should be on medical therapy to control hypertension. Blood pressure in this group of patients is difficult to control and they usually require multiple antihypertensives from different drug classes. 1 As the hypertension is mediated by activation of renin–angiotensin axis, ACEIs or ARBs are frequently used. Starting therapy with these drugs in the setting of bilateral severe RAS, stenosis affecting a single kidney, stenosis with a contralateral atrophic kidney, or stenosis with advanced chronic kidney disease can precipitate acute deterioration of renal function. Caution should be administered in such instances with regular monitoring of renal function, as early withdrawal of the ACEIS/ARBs can halt and reverse the acute kidney injury. 3 ACEIs/ARBs have a mortality benefit in patients with ARAS probably due to mitigation of adverse cardiovascular effects of elevated angiotensin II. 3 4

Beta blockers, calcium channel blockers, and diuretics are other antihypertensive drug classes that are often combined with ACEIs/ARBs. The aim should be to achieve reasonable blood pressure control with the fewest number of drugs and minimum adverse effects. 16

Those with ARAS should be advised to stop smoking, and they are usually started on antiplatelet agents and statins to combat the high risk of adverse cardiovascular events. 3 13

## Revascularization

Nonrandomized studies done in the 1990s claimed that there was a benefit in revascularization for ARAS in terms of improving blood pressure control and stabilization of renal function. 17 More recent randomized clinical trials (RCTs) have produced evidence that contradict these findings. This had led to a more restricted approach for revascularization in patients with ARAS.

According to the RCT done by the Newcastle Renal Artery Stenosis Collaborative Group, who compared balloon angioplasty with medical therapy versus medical therapy alone for the control of hypertension in patients with ARAS, only a modest improvement of blood pressure was noted by the addition of angioplasty. This effect was only seen in a subgroup of patients with bilateral RAS at the cost of significant periprocedural complications. No patient was cured of hypertension after balloon angioplasty. 18 Another study was done by the Dutch Renal Artery Stenosis Intervention Cooperative Study group reported that addition of angioplasty had little benefit over medical management alone in controlling hypertension in ARAS. 19

The STAR study evaluated the effects of stenting of the renal arteries and the best medical therapy versus best medical therapy alone on the progression of renal impairment in patients with ARAS who had a creatinine clearance of <80 mL/min/1.73 m ^2^ . The best medical therapy included antihypertensive agents, aspirin, and a statin. The study concluded that stenting had no clear effect on the progression of renal impairment. 20 Once structural damage to the kidneys has set in, revascularization probably fails to reverse these effects or alters its progression. 16

ASTRAL study was another RCT with a much larger study population. Eight hundred and six patients with ARAS were randomized to revascularization (angioplasty ± stenting with 95% being stented) with medical therapy or medical therapy alone. The effects on renal function, blood pressure, adverse renal and cardiovascular outcomes, and mortality were studied and compared between the two groups. There was no significant benefit in revascularization with regard to these outcomes. There was a periprocedural complication rate of 9% in those who were revascularized which included two deaths and three toe/limb amputations. 21

The CORAL investigators randomized 947 patients with ARAS and hypertension or chronic kidney disease to angioplasty and stenting with medical therapy or medical therapy alone and studied the effects of revascularization on adverse cardiovascular and renal events. The conclusion of the investigators was that renal artery stenting did not have a significant benefit over medical therapy alone with regard to the clinical end points evaluated. 17

The expected outcome after revascularization for ARAS is better control of hypertension or a “cure” of hypertension. However, according to the current evidence from randomized clinical trials these outcomes are not achieved despite successful revascularization. Probably, a majority with RAS and hypertension have essential hypertension and all with RAS and hypertension should not be lumped as having “renovascular hypertension.” 16 So recognition of those with true hypertension caused by atherosclerotic narrowing of their renal arteries and selective revascularization of these patients would be the key. At the present, the standard investigations used for diagnosis of RAS do not accurately identify the functional significance of these lesions or predict the response to revascularization. 1

The solution to this problem would be a shift in focus that concentrates on the physiological significance of the stenosis rather than its anatomy. Pressure gradients across the lesion have been studied but they do not correlate well with the severity of hypertension or the serum creatinine level. 22 Novel techniques, such as the use of hyperemic pressure gradients and calculation of fractional flow reserve, may be better predictors of the hemodynamic significance of the stenosis and the response to revascularization. Renal hyperemia can be achieved by infusion of papaverine, dopamine, and acetylcholine. The evidence available for the use of these techniques in ARAS is very limited at present, and none of the large scale RCTs that are available have utilized these methods. 23

The results following revascularization for flash pulmonary edema in patients with ARAS are less confusing compared with revascularizations done for blood pressure control or ischemic nephropathy. Gray et al 24 performed renal artery angioplasty and stenting for 39 patients with ARAS and recurrent episodes of heart failure and flash pulmonary edema. All patients had >70% stenosis affecting bilateral renal arteries or a single functioning kidney. After revascularization, 77% did not require hospital admissions due to heart failure during the follow-up period.

## Indications for Revascularization

According to the 2017 guidelines published by the European Society of Cardiology, revascularization can be considered in patients with ARAS and sudden, unexplained pulmonary edema or heart failure. This is given as a class-IIb recommendation. 4 The indications for revascularization are much wider according to the AHA guidelines published in 2006, as follows: 13

- Hemodynamically significant ARAS is associated with resistant hypertension, malignant hypertension, accelerated hypertension, and hypertension associated with an unexplained unilateral small kidney, when the patient is intolerant of antihypertensive medication. (class IIa).
- Bilateral ARAS or ARAS in a single functioning kidney accompanied by progressive chronic renal impairment (class IIa).
- Unilateral ARAS and chronic renal impairment (class IIb).
- Hemodynamically significant RAS with recurrent, unexplained congestive heart failure, or episodes of flash pulmonary edema (class IIa).
- Patients with hemodynamically significant RAS and unstable angina (class IIb).
- Hemodynamically significant bilateral ARAS or ARAS in a single functioning kidney in an asymptomatic patient (class IIb).

AHA has defined the lesion as hemodynamically significant when the stenosis is >70% and when 50 to 70% stenosis accompanies a peak translesional pressure gradient of ≥20 mm Hg or a mean pressure gradient of ≥10 mm Hg. 13

## Options for Revascularization

Renal artery angioplasty and stenting is the preferred modality of revascularization over open revascularization at present. Stenting is associated with better technical success and lower restenosis compared with balloon angioplasty alone. 3 Endovascular techniques are rapidly evolving and studies using drug-eluting stents have reported lower restenosis rates when compared with bare metal stents. 25

Cherr et al 26 reported a large series of patients with ARAS who were managed surgically. Aortorenal bypass, splanchnorenal bypass, renal artery reimplantation, and endarterectomies were the types of operations performed for revascularization of the kidneys. The perioperative mortality was 4.6% and the morbidity was 16%. The main perioperative complications were myocardial infarction, stroke, arrhythmia, pneumonia, and renal impairment.

In the current endovascular era, open surgery is reserved when percutaneous intervention fails, for patients with complex renal artery anatomy, and for those who need concomitant open surgery for aortic pathologies such as an aneurysm. 4

## Conclusion

All with ARAS should be on medical therapy to control the blood pressure. Antiplatelet medications and statins are also given, as these patients have a high risk of adverse cardiovascular events. The indications for revascularization are less clear and available RCTs have their limitations. Further studies are needed to clearly identify those who will benefit from revascularization. Until more robust evidence emerges, it is rational to follow existing guidelines.
